# Role of the Promoter Polymorphism IL-6 −174G/C in Dermatomyositis and Systemic Lupus Erythematosus

**DOI:** 10.1155/2013/315365

**Published:** 2013-09-11

**Authors:** Maria Hristova, Lyubomir Dourmishev, Zornitsa Kamenarska, Svetla Nikolova, Radka Kaneva, Anton Vinkov, Marta Baleva, Daniela Monova, Vanio Mitev

**Affiliations:** ^1^Department of Clinical Laboratory and Clinical Immunology, Medical University-Sofia, 1 Georgi Sofijski Street, 1431 Sofia, Bulgaria; ^2^Department of Dermatology and Venereology, Medical University-Sofia, 1 Georgi Sofijski Street, 1431 Sofia, Bulgaria; ^3^Molecular Medicine Center, Medical University-Sofia, 2 Zdrave Street, 1431 Sofia, Bulgaria; ^4^Department of Medical Chemistry and Biochemistry, Medical University-Sofia, 2 Zdrave Street, 1431 Sofia, Bulgaria; ^5^28 Diagnostic and Consultative Center-Sofia, 1 Iliya Beshkov Street, 1592 Sofia, Bulgaria; ^6^Department of Nephrology, Ministry of Interior Hospital, 79 Skobelev Boulevard, 1606 Sofia, Bulgaria

## Abstract

The promoter polymorphism −174G/C within the interleukin-6 gene (IL-6) has been reported to have a functional importance through the modulation of IL-6 gene expression in vitro and in vivo. IL-6 is thought to play an important role in autoimmune diseases and the effect of its receptor inhibitor—tocilizumab—has been recently studied. The aim of this case-control study was to investigate the association between the interleukin-6 −174G/C single nucleotide polymorphism and the susceptibility to dermatomyositis (DM) and systemic lupus erythematosus (SLE) in Bulgarian patients. Altogether, 87 patients—52 with SLE and 35 with DM—as well as 80 unrelated healthy controls were included in this study. All of them were analyzed by restriction fragment length polymorphism analysis (RFLP). The GG genotype and the G allele appeared to be associated with SLE, especially in women. None of the genotypes showed an association with DM. However, the G allele appeared to be associated with muscle weakness and it is a risk factor for elevated muscle enzymes. Our results indicate that IL-6 −174G/C polymorphism might be associated with the susceptibility to SLE especially in women. Although it is not associated with DM, it seems that IL-6 −174G/C polymorphism could modulate some clinical features in the autoimmune myopathies.

## 1. Introduction

Dermatomyositis (DM) and systemic lupus erythematosus (SLE) are diseases of unknown etiology. However, the dysregulation of cytokine production or action is thought to have an important role in their development [1]. The cytokine secretion was found to be under genetic control [2]. In our previous study, we have found association between TNF-*α* polymorphisms and the etiology of DM and SLE in Bulgarian patients [3].

TNF-*α* and IL-1 can induce IL-6 and IL-6 induces B-cell differentiation to plasma cells, hyperactivity, and secretion of antibodies and also promotes T-cell proliferation, cytotoxic T-cell differentiation, and local inflammation. According to Linker-Israeli et al. [4], elevated plasma levels of IL-6 messenger-RNA and protein could be detected in SLE patients. Serum TNF-*α* and IL-6 levels were reported to be sensitive markers for SLE activity [5, 6]. IL-6 is also considered to play a role in the development of DM, since it leads to reduced myogenesis [7]. Hagiwara et al. [8] have found that patients with DM and polymyositis have an increased number of IL-6 secreting cells compared to controls. Bilgic et al. [9] suggest that IL-6 serum level is a candidate biomarker for disease activity in both adult and juvenile DM.

The G allele of the IL-6 −174G/C polymorphism is associated with higher IL-6 expression [10]. The presence of the G allele has been associated with poor outcome in a variety of diseases including coronary artery disease [11], more severe manifestations of the Sjögren's syndrome [12] and systemic sclerosis [13], increased risk of juvenile rheumatoid arthritis [10], and growth retardation of children with Crohn's disease [14]. It was recently found that the IL-6 −174G/C gene promoter polymorphism predicts therapeutic response to TNF-*α* blockers [15].

The objective of our pilot study was to determine whether the IL-6 −174G/C polymorphism is a risk factor for the development of adult DM and SLE in Bulgarian patients and to define its contribution to the increased risk.

## 2. Materials and Methods

### 2.1. Clinical Material

Thirty-five patients with dermatomyositis who met the criteria of Bohan and Peter [16, 17] and Targoff et al. [18] and fifty-two with systemic lupus erythematosus who met the American College of Rheumatology (ACR) criteria were included in this study. The clinical and demographic data are presented in Table 1. In the DM group, 22 patients were females and 13 males. The mean age was 52 with a range of 18–82 years. In five patients, DM was associated with malignancy (breast and gastric carcinomas, myeloma and seminoma). In the SLE group, 43 were females and 9 males. The mean age was 40 with a range of 15–78 years. All of them showed renal involvement to a different extent. The patients have been followed for a mean of 10 years at the Department of Dermatology and Venereology, Medical University-Sofia, at the Department of Nephrology, Medical University-Sofia and at the Department of Nephrology, Ministry of Interior Hospital-Sofia.

The control group consisted of 80 anonymous healthy volunteers who did not show any clinical or laboratory signs of autoimmune skin diseases, as well as kinship with patients suffering from autoimmune skin diseases. They were randomly selected from the Biobank of the Molecular Medicine Center and the National Genetic Laboratory as to match the patients in age, gender, and ethnicity. The mean age was 45 ± 13.8 with a range of 19–76.

### 2.2. Genetic Analysis

The scientific investigation presented in this paper has been carried out in accordance with The Code of Ethics of the World Medical Association (Declaration of Helsinki) for experiments involving humans. The study was approved by the local ethics committee at the Medical University—Sofia. All participants signed an informed consent and venous blood was drawn for DNA isolation. Genomic DNA was extracted from the peripheral blood with the Chemagen DNA purification kit, using Chemagic Magnetic Separation Module I (Chemagen AG).

The analysis of IL-6 −174G/C polymorphism was performed as previously described by Libra et al. [19].

### 2.3. Statistical Analysis

Allele and genotype frequencies were compared between DM and SLE cases and controls, using Fisher's exact test to calculate *P* values for 2 × 2 tables. Where significant, data were expressed as *P* value and odds ratios (OR) with exact 95% confidence intervals (CI). Bonferroni correction was applied with a threshold significance level of 0.01. The test for Hardy-Weinberg equilibrium was done by *χ* ^2^ statistics.

## 3. Results

The single nucleotide polymorphism (SNP) was found to be in Hardy-Weinberg equilibrium. The observed allele and genotype frequencies of the IL-6 −174G/C polymorphism among the patients with DM and SLE and the healthy controls are summarized in Table 1.

The frequency of the GG genotype was higher among SLE patients (55.8%) compared to controls (40.0%) and it is associated with an increased OR (*P* = 0.055, after Bonferroni correction *P* = 0.28, OR 1.9; 95% CI 0.9–3; Table 2). Stratification by gender led to a stronger association of the GG genotype with SLE in women (*P* = 0.008, after Bonferroni correction *P* = 0.04, OR 2.9, 95% CI 1.3–6.6; Table 3). No difference was found in the genotype distribution between DM patients and controls. Stratification by gender did not lead to any statistically significant results.

The G allele showed an association with SLE (*P* = 0.01, after Bonferroni correction *P* = 0.05, OR 1.9, 95% CI 1.1–3.2; Table 2). Stratification by gender led to a stronger association of the G allele with SLE in women (*P* = 0.0002, after Bonferroni correction *P* = 0.001, OR = 3, 95% CI 1.6–5.4; Table 3). No significant increase of the G allele was found in the DM group versus controls (Table 2), and stratification by gender did not lead to any statistically significant results (Table 3).

No association was found between the −174G/C polymorphism and SLE clinical features (Table 4).

An association was found between the GG and GC genotypes and the G allele with muscle weakness (GG + GC—*P* = 0.012, after Bonferroni correction *P* = 0.06, OR 11.5, 95% CI 1.7–77.2; G—*P* = 0.023, after Bonferroni correction *P* = 0.12, OR 4.2, 95% CI 1.2–15; Table 5) and the elevated muscle enzymes (GG + GC—*P* = 0.013, after Bonferroni correction *P* = 0.065, OR 8.5, 95% CI 1.5–49.5; G—*P* = 0.054, after Bonferroni correction *P* = 0.27, OR 2.5, 95% CI 0.9–6.5; Table 5) in DM patients.

## 4. Discussion

From all polymorphisms within the IL-6 gene, the promoter polymorphism −174G/C seems to be of greatest significance for the development of autoimmune diseases. However, the results published so far in the literature are rather conflicting.

In our study, no association was found between the GG genotypes and DM but its frequency was higher among the SLE patients compared to the controls and it is associated with an increased OR. Stratification by gender led to a stronger association of the GG genotype with SLE in women. The association remained statistically significant after the application of Bonferroni correction. The GG genotype was earlier found to be increased in Egyptian [20] and Malaysian [21] patients with SLE but no significant differences in the genotype frequencies were found between German patients and healthy controls [22]. There were no significant differences in the genotype frequencies between Portuguese [23] and Chinese [24] patients with SLE either. Probably such differences could be explained with population variations [25]. In our study, no marked increase of the GC genotype was observed among the controls but such elevation was earlier found among Egyptian [20] and Malaysian [21] controls. Chua et al. [21] suggested that the C allele might have a masking effect on the G allele when both alleles are present in heterozygous individuals. However, they have not observed any significant association of the homozygous C allele with the onset of SLE or with protection from the disease.

In our study, the G allele showed an association with SLE. Stratification by gender led to a stronger association of the G allele with SLE in women. The associations remained significant after the application of Bonferroni correction. No significant increase of the G allele was found in the DM group versus controls, and stratification by gender did not lead to any statistically significant results. Earlier studies showed no significant differences in allele frequencies between German SLE patients and healthy controls [22]. There were no significant differences in the allele frequencies between Portuguese [23] and Chinese [24] patients with SLE either. However, our results are in line with the results of a recent meta-analysis which proved that the −174G allele is associated with SLE mainly in the European populations and less with SLE in the Asian ones [26]. 

Earlier study of the relationship between −174G/C polymorphism and clinical manifestations and laboratory parameters showed an association between the G allele and the presence of anti-nuclear antibodies in all patients and rash and hematuria in male patients with SLE [27]. By comparing patients with various disease manifestations, Schotte et al. [22] found an association of the −174G allele with discoid skin lesions and anti-histone antibodies. Hamdy et al. [20] reported a statistically significant increase of the GG genotype with pulmonal affection, nephritis, and arthritis. The SLE patients with GG genotype showed significantly higher frequencies and increased risk of constitutional manifestations at disease onset, photosensitivity, hematological disorders, and positivity of ANA and anti-dsDNA [27]. Quite surprisingly, Santos et al. [23] found the −174CC genotype but not the GG genotype to be independently associated with renal disease and the C allele with the presence of irreversible damage. Huang et al. [24] did not detect any association between the IL-6 genotype and clinical or laboratory profiles in SLE patients. Similarly to the results of Huang et al. [24], we found no relationship between the −174G/C polymorphism and ACR criteria. Although the statistical significance was lost when Bonferroni correction was applied, an increased frequency of the GG and GC genotypes and the G allele was found in DM patients with muscle weakness and elevated muscle enzymes which is in line with the role of IL-6 as a myokine. Our results confirm other authors' findings that the elevated levels of IL-6 lead to muscle weakness [28, 29]. To the best of our knowledge, this is the first study that investigates the IL-6 −174G/C polymorphism in patients with DM.

The major limitation of our study is the small sample size which could affect the statistical power of the results. Obviously, more studies in a larger cohort are needed to clarify the role of this polymorphism for the susceptibility and clinical manifestations of SLE and DM.

In conclusion, our results show that the IL-6 −174G/C polymorphism might be associated with SLE mainly in women. The GG genotype and G allele might be associated with DM clinical features.

## Figures and Tables

**Table 1:** Demographic and clinical data.

Disease	DM		SLE	
Demographic parameters	Female/male	22/13	Female/male	43/9
	Age, mean ± SD years	52 ± 14.7	Age, mean ± SD years	40 ± 12.4

Clinical parameters	Cutaneous disease	27 (77.1%)	Malar rash	34 (65.4%)
	Muscle weakness	28 (80.0%)	Discoid rash	9 (17.3%)
	Elevated muscle enzymes	19 (54.3%)	Arthritis	34 (65.4%)
	EMG findings	20 (57.1%)	Oral ulcer	3 (5.8%)
	Photosensitivity	21 (60.0%)	Photosensitivity	30 (57.7%)
	Immunological disease	9 (25.7%)	Serositis	11 (21.2%)
			Renal disease	52 (100%)
			Neurological disease	10 (19.2%)
			Haematological disease	20 (38.5%)
			Immunological disease	32 (61.5%)
			ANA	36 (69.2%)

**Table 2:** Genotype and allele frequencies of IL-6 −174G/C polymorphism in dermatomyositis and systemic lupus erythematosus patients and controls.

Genotype	DM	SLE	Controls
*N* = number of patients	*N* = 35	*N* = 52	*N* = 80
−174G/C			
GG	14 (40.0%)	29 (55.8%)	32 (40.0%)
GC	11 (31.4%)	13 (25.0%)	21 (26.3%)
CC	10 (28.6%)	10 (19.2%)	27 (33.7%)
G	55.7%	68.3%	53.1%
C	44.3%	34.6%	46.9%
*P* value	NS*	**G**—***P* = 0.01**	

*Not significant.

**Table 3:** Allele and genotype frequency of the IL-6 −174G/C polymorphism in females with dermatomyositis and systemic lupus erythematosus and in controls.

Genotype	DM	SLE	Controls
*N* = number of females	*N* = 22	*N* = 43	*N* = 58
−174G/C			
GG	8 (36.4%)	26 (60.5%)	20 (34.5%)
GC	6 (27.2%)	10 (23.2%)	14 (24.1%)
CC	8 (36.4%)	7 (16.3%)	24 (41.4%)
G	50.0%	72.1%	46.6%
C	50.0%	27.9%	53.4%
*P* value	NS*	**GG—0.008**; **G—0.0002**	

*Not significant.

**Table 4:** Comparison between the genotypes and ACR criteria.

Genotype	GG (*n* = 29)	GC (*n* = 13)	CC (*n* = 10)	*P* value
Malar rash	20 (69.0%)	5 (38.5%)	9 (90.0%)	NS*
Discoid rash	6 (20.7%)	3 (23.1%)	0 (0.0%)	NS
Photosensitivity	17 (58.6%)	8 (61.5%)	5 (50.0%)	NS
Oral ulcer	1 (3.4%)	2 (15.4%)	0 (0.0%)	NS
Arthritis	18 (56.5%)	9 (69.2%)	7 (70.0%)	NS
Serositis	6 (20.7%)	4 (30.8%)	1 (10.0%)	NS
Renal disease	29 (100.0%)	13 (100.0%)	10 (100.0%)	—
Neurological disease	6 (26.1%)	2 (15.4%)	2 (20.0%)	NS
Haematological disease	11 (37.9%)	4 (30.8%)	5 (50.0%)	NS
Immunological disease (anti-dsDNA, anti-Sm, and antiphospholipid Ab)	20 (69.0%)	6 (46.2%)	6 (60.0%)	NS
ANA	22 (75.9%)	7 (53.8%)	7 (70.0%)	NS

*Not significant.

**Table 5:** Comparison between the genotypes and DM clinical parameters.

Genotype	GG (*n* = 14)	GC (*n* = 11)	CC (*n* = 10)	*P* value
Muscle weakness	12 (85.7%)	11 (100.0%)	5 (50.0%)	**GG + GC—*P* = 0.012, G—*P* = 0.023**
Elevated muscle enzymes	8 (57.1%)	9 (81.8%)	2 (20.0%)	**GG + GC—*P* = 0.013**, **G—*P* = 0.054**
EMG findings	7 (50.0%)	8 (72.7%)	5 (50.0%)	NS*
Cutaneous disease	11 (78.6%)	9 (81.8%)	7 (70.0%)	NS
Photosensitivity	10 (71.4%)	6 (54.5%)	5 (50.0%)	NS
Immunological disease (anti-myositis-specific antibodies)	3 (21.4%)	4 (36.4%)	2 (20.0%)	NS

*Not significant.

## References

[1] O’Shea JJ, Ma A, Lipsky P (2002). Cytokines and autoimmunity. *Nature Reviews Immunology*.

[2] Pawlik A, Baskiewicz-Masiuk M, Machalinski B, Gawronska-Szklarz B (2006). Association of cytokine gene polymorphisms and the release of cytokines from peripheral blood mononuclear cells treated with methotrexate and dexamethasone. *International Immunopharmacology*.

[3] Dourmishev L, Kamenarska Z, Hristova M, Dodova R, Kaneva R, Mitev V (2012). Association of TNF-*α* polymorphisms with adult dermatomyositis and systemic lupus erythematosus in Bulgarian patients. *International Journal of Dermatology*.

[4] Linker-Israeli M, Wallace DJ, Prehn J (1999). Association of IL-6 gene alleles with systemic lupus erythematosus (SLE) and with elevated IL-6 expression. *Genes and Immunity*.

[5] Sabry A, Sheashaa HA, El-Husseini A (2006). Proinflammatory cytokines (TNF-*α* and IL-6) in Egyptian patients with SLE: Its correlation with disease activity. *Cytokine*.

[6] Sabry A, Elbasyouni SR, Sheashaa HA (2006). Correlation between levels of TNF-alpha; and IL-6 and hematological involvement in SLE Egyptian Patients with lupus nephritis. *International Urology and Nephrology*.

[7] Langen RCJ, Schols AMWJ, Kelders MCJM, van der Velden JLJ, Wouters EFM, Janssen-Heininger YMW (2002). Tumor necrosis factor-*α* inhibits myogenesis through redox-dependent and -independent pathways. *American Journal of Physiology*.

[8] Hagiwara E, Adams EM, Plotz PH, Klinman DM (1996). Abnormal numbers of cytokine producing cells in patients with polymyositis and dermatomyositis. *Clinical and Experimental Rheumatology*.

[9] Bilgic H, Ytterberg SR, Amin S (2009). Interleukin-6 and type I interferon-regulated genes and chemokines Mark disease activity in dermatomyositis. *Arthritis and Rheumatism*.

[10] Fishman D, Faulds G, Jeffey R (1998). The effect of novel polymorphisms in the interleukin-6 (IL-6) gene on IL-6 transcription and plasma IL-6 levels, and an association with systemic- onset juvenile chronic arthritis. *The Journal of Clinical Investigation*.

[11] Humphries SE, Luong LA, Ogg MS, Hawe E, Miller GJ (2001). The interleukin-6 −174G/C promoter polymorphism is associated with risk of coronary heart disease and systolic blood pressure in healthy men. *European Heart Journal*.

[12] Hulkkonen J, Pertovaara M, Antonen J, Pasternack A, Hurme M (2001). Elevated interleukin-6 plasma levels are regulated by the promoter region polymorphism of the IL6 gene in primary Sjögren’s syndrome and correlate with the clinical manifestations of the disease. *Rheumatology*.

[13] Sfrent-Cornateanu R, Mihai C, Balan S, Ionescu R, Moldoveanu E (2006). The IL-6 promoter polymorphism is associated with disease activity and disability in systemic sclerosis. *Journal of Cellular and Molecular Medicine*.

[14] Sawczenko A, Azooz O, Paraszczuk J (2005). Intestinal inflammation-induced growth retardation acts through IL-6 in rats and depends on the −174 IL-6 G/C polymorphism in children. *Proceedings of the National Academy of Sciences of the United States of America*.

[15] di Renzo L, Bianchi A, Saraceno R (2012). −174G/C IL-6 gene promoter polymorphism predicts therapeutic response to TNF-*α* blockers. *Pharmacogenetics and Genomics*.

[16] Bohan A, Peter JB (1975). Polymyositis and dermatomyositis I. *The New England Journal of Medicine*.

[17] Bohan A, Peter JB (1975). Polymyositis and dermatomyositis II. *The New England Journal of Medicine*.

[18] Targoff IN, Miller FW, Medsger TA, Oddis CV (1997). Classification criteria for the idiopathic inflammatory myopathies. *Current Opinion in Rheumatology*.

[19] Libra M, Signorelli SS, Bevelacqua Y (2006). Analysis of G(−174)C IL-6 polymorphism and plasma concentrations of inflammatory markers in patients with type 2 diabetes and peripheral arterial disease. *Journal of Clinical Pathology*.

[20] Hamdy E, Afify RA, Kamal A, Abass D, Mahmoud M (2012). IL-6 promoter polymorphism (−174G/C) and systemic lupus erythematosus. *Comperative Clinical Pathology*.

[21] Chua KH, Kee BP, Tan SY, Lian LH (2009). Interleukin-6 promoter polymorphisms (−174G/C) in Malaysian patients with systemic lupus erythematosus. *Brazilian Journal of Medical and Biological Research*.

[22] Schotte H, Schlüter B, Rust S, Assmann G, Domschke W, Gaubitz M (2001). Interleukin-6 promoter polymorphism (−174G/C) in Caucasian German patients with systemic lupus erythematosus. *Rheumatology*.

[23] Santos MJ, Fernandes D, Capela S, da Silva JC, Fonseca JE (2011). Interleukin-6 promoter polymorphism −174G/C is associated with nephritis in Portuguese Caucasian systemic lupus erythematosus patients. *Clinical Rheumatology*.

[24] Huang C-M, Huo A-P, Tsai C-H, Chen C-L, Tsai F-J (2006). Lack of association of interleukin-6 and interleukin-8 gene polymorphisms in Chinese patients with systemic lupus erythematosus. *Journal of Clinical Laboratory Analysis*.

[25] Borinskaya SA, Gureev AS, Orlova AA (2013). Allele frequency distributions of *−174G/C* polymorphism in regulatory region of interleukin 6 gene (*IL6*) in Russian and worldwide populations. *Russian Journal of Genetics*.

[26] Lee YH, Lee HS, Choi SJ, Ji JD, Song GG (2012). The association between interleukin-6 polymorphisms and systemic lupus erythematosus: a meta-analysis. *Lupus*.

[27] Godarzi EM, Sarvestani EK, Aflaki E, Amirghofran Z (2011). Interleukin-6 gene polymorphism in Iranian patients with systemic lupus erythematosus. *Clinical Rheumatology*.

[28] Schaap LA, Pluijm SMF, Deeg DJH, Visser M (2006). Inflammatory markers and loss of muscle mass (sarcopenia) and strength. *The American Journal of Medicine*.

[29] Haddad F, Zaldivar F, Cooper DM, Adams GR (2005). IL-6-induced skeletal muscle atrophy. *Journal of Applied Physiology*.

